# A National Study Exploring the Association Between Fluoride Levels and Dental Fluorosis

**DOI:** 10.1001/jamanetworkopen.2023.18406

**Published:** 2023-06-23

**Authors:** Man Hung, Eric S. Hon, Amir Mohajeri, Hyma Moparthi, Teresa Vu, Jason Jeon, Martin S. Lipsky

**Affiliations:** 1 College of Dental Medicine, Roseman University of Health Sciences, South Jordan, Utah; 2 Division of Public Health, University of Utah, Salt Lake City; 3 College of Social Work, University of Utah, Salt Lake City; 4 College of Education, University of Utah, Salt Lake City; 5 George E. Wahlen Department of Veterans Affairs Medical Center, Salt Lake City, Utah; 6 Department of Economics, University of Chicago, Chicago, Illinois; 7 Portland State University Institute on Aging, Portland, Oregon

## Abstract

**Importance:**

While the effects of fluoride on health have been widely researched, fewer high-quality studies examine the association of fluoride levels in water and dental fluorosis.

**Objective:**

To investigate the association between fluoride exposure from drinking water and dental fluorosis.

**Design, Setting, and Participants:**

This cross-sectional study used the 2013-2014 and 2015-2016 National Health and Nutrition Examination Survey (NHANES) data (January 1, 2013, through December 31, 2016). NHANES uses a complex sampling technique to develop nationally representative sample estimates of the US population that consists of interviews and physical assessments. Children and adolescents aged 6 to 15 years were included because NHANES contains their data for all 3 forms of fluoride measures: plasma fluoride levels, water levels of fluoride, and dietary fluoride supplementation. Data were analyzed from January 1 to April 30, 2023.

**Exposures:**

Water and plasma fluoride levels were measured by laboratory personnel. Dietary fluoride supplement data were self-reported.

**Main Outcomes and Measures:**

The Dean’s Fluorosis Index was used to evaluate fluorosis status for each tooth. The dental fluorosis severity value was based on the second most affected tooth. Independent variables included plasma and water fluoride concentrations and dietary fluoride supplementation. An independent samples *t* test was used to compare fluoride exposures between groups, and Pearson correlation assessed the association between plasma and water fluoride levels. To assess whether fluoride exposures were associated with dental fluorosis, logistic regression analyses were conducted.

**Results:**

There were 1543 participants in the 2013-2014 NHANES cycle (weighted proportion male, 51.9%; mean [SD] age, 11.0 [2.7] years) and 1452 in the 2015-2016 cycle (weighted proportion male, 52.6%; mean [SD] age, 11.1 [2.8] years). A weighted 87.3% exhibited some degree of fluorosis in the 2013-2014 cycle and 68.2% in the 2015-2016 cycle. After adjusting for covariates in the 2015-2016 cycle, both higher water and plasma fluoride concentrations were associated with higher odds of dental fluorosis: adjusted odds ratios [AORs], 2.378 (95% CI, 1.218-5.345]) for water fluoride and 1.568 (95% CI, 1.038-2.499) for plasma fluoride. Fluoride supplements were not significantly associated with dental fluorosis: AOR, 0.741 (95% CI, 0.367-1.408).

**Conclusions and Relevance:**

The findings of this cross-sectional study suggest that exposure to higher concentrations of fluoride in water and having higher plasma levels of fluoride were associated with a greater risk of dental fluorosis. Further research can help policy makers develop policies that balance substantial caries prevention with the risk of dental fluorosis.

## Key Points

**Question:**

What is the association between systemic fluoride exposure and dental fluorosis?

**Findings:**

This cross-sectional study of 2995 children and adolescents found that higher fluoride levels in water and plasma were associated with dental fluorosis.

**Meaning:**

These findings suggest that public health policy related to water fluoride levels and fluoridation should consider balancing caries prevention with dental fluorosis risk.

## Teaser

This cross-sectional study uses data from 2 cycles of the National Health and Nutrition Examination Survey to explore associations between fluoride exposures and dental fluorosis in a nationally representative sample of US children and adolescents.

## Introduction

When given in an appropriate amount, fluoride helps prevent dental caries by hardening enamel and enhancing tooth remineralization. It does so by promoting the tooth’s calcium and phosphate ion remineralization process in rebuilding a new surface on existing crystal remnants. These remineralized crystals incorporate fluoride and are more acid-resistant than the original enamel mineral, which further protects enamel from decay.^1^

However, excess fluoride during tooth development can cause dental fluorosis, which are visible changes on an erupting tooth’s enamel surface. Depending on the amount, duration, and timing of fluoride intake, fluorosis can appear in varying forms in both primary and permanent teeth. Mild cases include scattered white flecks that are barely noticeable, while more moderate and severe cases can cause brown stains and rough, pitted surfaces.^2^ Dental fluorosis is only considered a risk to children 8 years or younger because permanent teeth are fully developed after this age.^3^

The oral health benefits of fluoride are validated by epidemiologic evidence. Several systematic reviews^4,5,6,7,8,9^ support both the clinical effectiveness and cost-effectiveness of fluoridation. Drinking fluoridated water reduces tooth decay by approximately 25% in children and adults,^3,10^ and every dollar spent on fluoridation yields as much as $32 in reduced dental care expenses.^11^

The only widely recognized harm from fluoridation is dental fluorosis. Most cases of dental fluorosis are mild, and, other than cosmetic changes, dental fluorosis does not cause symptoms or harm. In determining fluoride recommendations, policy makers sought to balance dental caries prevention while limiting the risk of dental fluorosis and other potential health harms.^12^ However, despite an abundance of studies related to fluoride,^13^ fewer high-quality studies examine the association of water fluoride levels with the prevalence of dental fluorosis.^10,14^

This study used a nationally representative sample of children in the US to explore associations between fluoride exposures and dental fluorosis. The findings contribute to the literature by updating earlier research about dental fluorosis and can help policy makers and health care professionals to balance the risks and benefits of fluoride.

## Methods

### Study Design

This study used a cross-sectional design, analyzing data from the National Health and Nutrition Examination Survey (NHANES). Study reporting followed the Strengthening the Reporting of Observational Studies in Epidemiology (STROBE) guideline for cross-sectional studies. The National Center for Health Statistics created public use files, and NHANES for the 2013-2014 and 2015-2016 cycles was approved by the National Center for Health Statistics Research Ethics Review Board, which waived the need for informed consent for the use of publicly available data.

### Data Source

The study used data from NHANES from the 2013-2014 and 2015-2016 cycles (January 1, 2013, through December 31, 2016). These were different participants from the 2 cycles of data, which represented the cross-sectional design of the study. NHANES is a survey aimed at determining the health and nutritional status of all US residents, including adults and children. The survey consists of both interviews and physical assessments. Health interviews are performed at the respondent’s home, and health assessments are performed in mobile examination centers (MECs) that travel across the country. Data collection for this continuous program began in 1999 with a nationally representative sample of approximately 5000 persons each cycle. More details regarding NHANES study procedures can be found elsewhere.^15^

### Inclusion Criteria

Parents of participants aged 6 to 15 years were asked whether the participants received fluoride supplements. This age range was included in the analyses because NHANES contained data of their fluoride measurements, dental fluorosis assessments, and covariates needed for this study. A sample size of 1543 participants were in the 2013-2014 cycle of data and 1452 in the 2015-2016 cycle, for a total sample size of 2995 participants without any missing data on the selected variables for this study.

### Measures

#### Outcome Measures

Outcome measures were evaluated at the NHANES MECs by laboratory personnel and dental examiners. NHANES dental examiners used the Dean’s Fluorosis Index (DFI) to evaluate fluorosis status for each tooth with the following categories: normal tooth (translucent, smooth, glossy, pale creamy white [DFI = 0]), questionable tooth (slight aberrations, a few white spots [DFI = 0.5]), very mild fluorosis (<25% of tooth has small, white areas [DFI = 1.0]), mild fluorosis (25%-50% of the tooth has white areas [DFI = 2.0]), moderate fluorosis (≥50% of the tooth with all surfaces involved, with or without brown stains [DFI = 3.0]), and severe fluorosis (all enamel is involved and has discrete or confluent pitting [DFI = 4.0]).^16^ The dental fluorosis severity value was based on the second most affected tooth. The person’s status would be determined by the less affected tooth if the 2 most affected teeth were not equally affected (NHANES 2016). In this study, the dental fluorosis variable was dummy coded, where DFI ≤ 0.5 was assigned to participants with no fluorosis and DFI ≥ 1.0 to those with fluorosis.

#### Independent Variables

This study used 2 continuous variables (plasma fluoride concentration and water fluoride concentration) and 1 binary variable (fluoride in supplement form) as independent variables. Self-reported responses to the following question were used to assess the fluoride supplement: Have you ever received prescription fluoride drops or fluoride tablets (yes or no)? Both plasma and water fluoride concentrations were analyzed and recorded at the MECs by laboratory personnel. To measure plasma fluoride concentration, each sample underwent measurement twice using the ion-specific electrode and hexamethyldisiloxane method, and then the mean of the 2 measurements was calculated. Fluoride concentration in water samples was also measured twice using an ion-specific electrode,^3^ and then the mean was calculated. Based on the US Public Health Service–recommended water fluoride concentration of 0.70 mg/L,^12^ fluoride levels in the water were categorized as 0.30 mg/L or less (reference level), 0.31 to 0.50 mg/L (level 1), 0.51 to 0.70 mg/L (level 2), and greater than 0.70 mg/L (level 3) in this study. Plasma fluoride was also categorized into 4 levels, which were 0.30 μmol/L or less (reference level), 0.31 to 0.40 μmol/L (level 1), 0.41 to 0.50 μmol/L (level 2), and greater than 0.50 μmol/L (level 3).

#### Covariates

This study adjusted for covariates, which included sociodemographic factors: the child’s age (6-11 years [hereinafter referred to as children] or 12-15 years [hereinafter referred to as adolescents]), the child’s sex (male or female), the child’s race or ethnicity (Mexican American, non-Hispanic Asian, non-Hispanic Black, non-Hispanic White, non-Mexican Hispanic, or other [all non-Hispanic individuals of >1 race]), family educational level (<9th grade, 9th-11th grades, high school graduate or attainment of a General Educational Development certificate, some college, or college graduate or above), the season of sample collection (November 1 through April 30 or May 1 through October 31), and the ratio of family income to the area poverty level. The family educational level refers to the educational level of the person who owns or rents the residence where the study participant resides.^17^

### Statistical Analysis

Data were analyzed from January 1 to April 30, 2023. Means and proportions were calculated for demographic variables and fluoride exposure using the unweighted data to reflect the survey sample characteristics and the weighted data to produce nationally representative estimates. Pearson correlation coefficients were computed between plasma fluoride concentration and water fluoride concentration. Independent samples *t* tests were used to explore differences in fluoride exposures across different groups. The associations between the oral health outcome (dental fluorosis) and fluoride exposures (water fluoride concentration, plasma fluoride concentration, and fluoride supplementation) were examined after controlling for age, sex, race and ethnicity, family educational level, ratio of family income to poverty, and period when the NHANES survey was administered using binary logistic regression. Regression models were run separately for the 2013-2014 data, the 2015-2016 data, and the combined data from 2013 to 2016. Separate analyses of the 2013-2014 data and the 2015-2016 data would allow for an examination of the 2015 recommendation by US Department of Health and Human Services (DHHS) to lower water fluoride concentrations.^12^ Additional regression models were run with incorporation of an interaction effect between the fluoride supplements and water fluoride levels controlling for covariates. The 95% CIs were reported. Statistical significance was defined as a 95% CI excluding 0 for differences and excluding 1 for ratios. All analyses were performed using SPSS, version 28 (IBM Corporation).

## Results

### Demographic Characteristics

Table 1 presents the demographic characteristics for each of the cycles. In total, there were 1543 participants aged 6 to 15 years representing 31 847 426 individuals in the 2013-2014 cycle (weighted proportions, 51.9% male and 48.1% female; mean [SD] age, 11.0 [2.7] years) and 1452 participants representing 32 340 232 individuals in the 2015-2016 cycle (weighted proportions, 52.6% male and 47.4% female; mean [SD] age, 11.1 [2.8] years). More than half were aged of 6 to 11 years (weighted 53.9% for 2013-2014 and 52.6% for 2015-2016). More than half of the population was non-Hispanic White (weighted 51.3% for 2013-2014 and 51.9% for 2015-2016) compared with Mexican American (weighted 17.7% for 2013-2014 and 15.5% for 2015-2016), non-Hispanic Asian (weighted 4.4% for both 2013-2014 and 2015-2016), non-Hispanic Black (weighted 14.1% for 2013-2014 and 12.9% for 2015-2016), non-Mexican Hispanic (weighted 7.1% for 2013-2014 and 10.0% for 2015-2016), and other (weighted 5.4% for 2013-2014 and 5.3% for 2015-2016). More than half of the data were collected from May 1 through October 31 (weighted 52.8% for 2013-2014 and 56.8% for 2015-2016), and most parents had some college education (weighted 31.0% for 2013-2014 and 34.4% for 2015-2016). In the 2013-2014 and 2015-2106 cycles, the mean (SD) ratios of family income to the poverty level were 2.3 (1.6) and 2.5 (1.5), respectively.

**Table 1.** Demographic Characteristics of National Health and Nutrition Examination Survey Participants From the 2013-2014 and 2015-2016 Cycles

Variable	Weighted No. (%)		Sample No. (%)	
	2013-2014 Cycle	2015-2016 Cycle	2013-2014 Cycle	2015-2016 Cycle
Age, mean (SD), y	11.0 (2.7)	11.1 (2.8)	10.6 (2.7)	10.6 (2.7)
Age categories^a^				
Children	17 179 395 (53.9)	17 025 031 (52.6)	934 (60.5)	894 (61.6)
Adolescents	14 668 030 (46.1)	15 315 201 (47.4)	609 (39.5)	558 (38.4)
Sex				
Male	16 518 229 (51.9)	17 013 628 (52.6)	800 (51.8)	739 (50.9)
Female	15 329 196 (48.1)	15 326 603 (47.4)	743 (48.2)	713 (49.1)
Race and ethnicity				
Mexican American	5 621 262 (17.7)	5 013 392 (15.5)	375 (24.3)	339 (23.3)
Non-Hispanic Asian	1 405 593 (4.4)	1 423 182 (4.4)	123 (8.0)	114 (7.9)
Non-Hispanic Black	4 505 358 (14.1)	4 178 481 (12.9)	405 (26.2)	315 (21.7)
Non-Hispanic White	16 342 985 (51.3)	16 797 154 (51.9)	387 (25.1)	392 (27.0)
Non-Mexican Hispanic	2 254 022 (7.1)	3 221 095 (10.0)	148 (9.6)	206 (14.2)
Other^b^	1 718 106 (5.4)	1 706 927 (5.3)	105 (6.8)	86 (5.9)
Family educational level				
<9th Grade	2 242 721 (7.2)	2 967 160 (9.4)	154 (10.2)	184 (13.0)
9th-11th Grade	3 604 823 (11.6)	3 863 995 (12.2)	239 (15.9)	196 (13.9)
High school graduate or GED	7 132 174 (23.0)	5 746 106 (18.2)	351 (23.3)	297 (21.0)
Some college	9 623 541 (31.0)	10 867 809 (34.4)	455 (30.2)	445 (31.4)
College graduate or higher	8 443 571 (27.2)	8 123 427 (25.7)	307 (20.4)	293 (20.7)
Ratio of family income to poverty, mean (SD)	2.3 (1.6)	2.5 (1.5)	1.9 (1.5)	2.1 (1.5)
Season of sample collection				
November 1 through April 30	15 045 091 (47.2)	13 969 779 (43.2)	801 (51.9)	674 (46.4)
May 1 through October 31	16 802 334 (52.8)	18 370 453 (56.8)	742 (48.1)	778 (53.6)

Abbreviation: GED, General Educational Development.

^a^ Children are aged 6 to 11 years; adolescents, aged 12 to 15 years.

^b^ Includes other race and all non-Hispanic persons reporting more than 1 race.

### Fluoride Variables

Table 2 contains descriptive statistics for water fluoride levels, plasma fluoride levels, and fluoride supplements. The mean (SD) fluoride content in the water was 0.56 (0.36) mg/L in 2013-2014 and 0.46 (0.38) mg/L in 2015-2016, both of which fell below the US Public Health Service recommended level of 0.7 mg/L. In 2015-2016, both the water fluoride level (mean [SD], 0.49 [0.40] mg/L) and plasma fluoride level (mean [SD], 0.38 [0.23] μmol/L) in children were higher than those in adolescents (Table 2). However, in 2013-2014, water fluoride levels for adolescents (mean [SD], 0.57 [0.39] mg/L) and plasma fluoride levels in children (mean [SD], 0.44 [0.27] μmol/L) were the highest. A higher percentage of adolescents received fluoride supplements than children in both periods (weighted 50.8% for 2013-2014 and 53.6% for 2015-2016) (Table 2).

**Table 2.** Comparison of Fluoride Exposure Across Groups

Fluoride exposure^a^	2013-2014 Cycle			2015-2016 Cycle		
	Weighted No. (%)	Mean (SD)	Mean difference (95% CI)	Weighted No. (%)	Mean (SD)	Mean difference (95% CI)
**Water fluoride concentration, mg/L**						
All	31 847 426 (100)	0.56 (0.36)	NA	32 340 232 (100)	0.46 (0.38)	NA
Children	17 179 395 (53.9)	0.55 (0.34)	−0.025 (−0.096 to 0.045)	17 025 031 (52.6)	0.49 (0.40)	0.067 (−0.001 to 0.134)
Adolescents	14 668 030 (46.1)	0.57 (0.39)		15 315 201 (47.4)	0.43 (0.34)	
November 1 through April 30	15 045 091 (47.2)	0.55 (0.34)	0.021 (−0.143 to 0.185)	13 969 779 (43.2)	0.41 (0.40)	−0.099 (−0.273 to 0.074)
May 1 through October 31	16 802 334 52.8	0.57 (0.39)		18 370 453 (56.8)	0.50 (0.35)	
**Plasma fluoride concentration, μmol/L**						
All	31 847 426 (100)	0.43 (0.48)	NA	32 340 232 (100)	0.35 (0.22)	NA
Children	17 179 395 (53.9)	0.44 (0.27)	0.026 (−0.022 to 0.074)	17 025 031 (52.6)	0.38 (0.23)	0.054 (0.030 to 0.079)
Adolescents	14 668 030 (46.1)	0.42 (0.69)		15 315 201 (47.4)	0.32 (0.20)	
November 1 through April 30	15 045 091 (47.2)	0.40 (0.24)	−0.004 (−0.081 to 0.071)	13 969 779 (43.2)	0.35 (0.27)	−0.002 (−0.055 to 0.051)
May 1 through October 31	16 802 334 (52.8)	0.47 (0.65)		18 370 453 (56.8)	0.35 (0.17)	
**Fluoride supplement use**						
All	460 6497 (100)	NA	NA	4 038 972 (100)	NA	NA
Children	2 266 345 (49.2)	NA	NA	1 874 254 (46.4)	NA	NA
Adolescents	2 340 152 (50.8)	NA		2 164 717 (53.6)	NA	
November 1 through April 30	1 486 602 (32.3)	NA	NA	2 216 167 (54.9)	NA	NA
May 1 through October 31	3 119 894 (67.7)	NA		1 822 805 (45.1)	NA	

Abbreviation: NA, not applicable.

^a^ Children are aged 6 to 11 years; adolescents, aged 12 to 15 years.

In the 2015-2016 cycle, plasma fluoride concentrations in May to October (mean [SD], 0.35 [0.17] μmol/L) were equivalent to those in November to April (mean [SD], 0.35 [0.27] μmol/L), even though water fluoride concentrations from May to October (mean [SD], 0.50 [0.35] mg/L) were higher than those in November to April (mean [SD], 0.41 [0.40] mg/L) (Table 2). Both the water fluoride level (mean [SD], 0.57 [0.39] mg/L) and plasma fluoride level (mean [SD], 0.47 [0.65] μmol/L) levels from May to October were higher than those from November to April in 2013-2014 (Table 2). Table 2 also reveals that the number of participants who used fluoride supplements increased from May through October (weighted 67.7%) compared with November through April (weighted 32.3%) in 2013-2014, while the number of participants receiving fluoride supplements from November to April (weighted 54.9%) was greater than the number of participants from May to October (weighted 45.1%) in 2015-2016. Fluoride concentrations in plasma and water were found to be positively correlated in both 2013-2014 (*r* = 0.18) and 2015-2016 (*r* = 0.35). In addition, water fluoride levels differed significantly between those who had ever used fluoride supplements and those who did not take any fluoride supplements in both periods (mean difference, 0.231 [95% CI, 0.147-0.315] for 2013-2014 and 0.171 [95% CI, 0.047-0.294] for 2015-2016) (Table 3).

**Table 3.** Comparison Between Water Fluoride Concentration and Fluoride Supplementation

Group	No. (%) of participants		Water fluoride concentration, mean (SD), mg/L	Mean difference (95% CI), mg/L
	Nonweighted	Weighted		
2013-2014 Cycle				
With fluoride supplement	172 (11.2)	4 606 497 (14.6)	0.35 (0.27)	0.231 (0.147-0.315)
Without fluoride supplement	1360 (88.8)	26 902 235 (85.4)	0.58 (0.35)	
2015-2016 Cycle				
With fluoride supplement	154 (10.7)	4 038 972 (12.6)	0.32 (0.33)	0.171 (0.047-0.294)
Without fluoride supplement	1285 (89.3)	27 997 977 (87.4)	0.49 (0.38)	

### Dental Health Status

In total, a weighted 87.3% of children and adolescents exhibited some degree of fluorosis (very mild, mild, moderate, and severe) in 2013-2014 and 68.2% in 2015-2016. Table 4 displays the unadjusted associations between fluoride exposures and fluorosis, demonstrating that higher water fluoride concentrations were associated with an increased risk of dental fluorosis in children and adolescents for both the 2013-2014 and 2015-2016 cycles. After adjusting for covariates in the 2015-2016 cycle, both higher water and plasma fluoride concentrations were still independently associated with higher odds of dental fluorosis (adjusted odds ratio [AOR], 2.378 [95% CI, 1.218-5.345] for water fluoride; AOR, 1.568 [95% CI, 1.038-2.499] for plasma fluoride) (Table 5). Fluoride supplements were not significantly associated with dental fluorosis (AOR, 0.741 [95% CI, 0.367-1.408]), with the observation that individuals taking supplements were exposed to lower water fluoride levels (Table 5), indicating a potential interaction effect between fluoride supplements and water fluoride levels. Further regression analyses found that the association of dental fluorosis with fluoride supplements varied by water fluoride levels when data were combined from both cycles; however, the interactions were not statistically significant at water fluoride levels of 0.31 to 0.50 mg/L (AOR, 1.12 [95% CI, 0.352-3.133]) and greater than 0.70 mg/L (AOR, 1.08 [95% CI, 0.253-3.223]) (eTable in Supplement 1).

**Table 4.** Unadjusted Association Between Fluoride Exposure and Fluorosis^a^

Fluoride exposure	Odds ratio (95% CI)		
	2013-2014 Cycle	2015-2016 Cycle	2013-2016 Cycles
Fluoride supplement			
None	1 [Reference]	1 [Reference]	1 [Reference]
Children	0.511 (0.252-1.037)	0.812 (0.441-1.496)	0.716 (0.456-1.125)
Adolescents	0.684 (0.324-1.441)	0.712 (0.329-01.542)	0.747 (0.459-1.041)
**Water fluoride level, mg/L**			
≤0.30	1 [Reference]	1 [Reference]	1 [Reference]
Children			
0.31-0.50	2.005 (1.098-3.660)	1.406 (0.470-4.205)	1.498 (0.699-3.210)
0.51-70	2.464 (1.060-5.729)	1.702 (0.713-4.062)	2.313 (1.281-4.179)
>0.70	2.465 (1.119-5.429)	2.362 (1.035-5.388)	2.520 (1.271-4.999)
Adolescents			
0.31-0.50	1.071 (0.314-3.653)	0.884 (0.244-3.197)	0.954 (0.569-2.580 )
0.51-0.70	1.305 (0.415-4.099)	2.397 (0.707-8.315)	2.372 (1.243-4.179)
>0.70	1.795 (0.603-5.344)	3.299 (1.351-8.053)	3.457 (1.472-5.077)
**Plasma fluoride level, μmol/L**			
≤0.30	1 [Reference]	1 [Reference]	1 [Reference]
Children			
0.31-0.40	0.964 (0.525-1.770)	1.110 (0.768-1.605)	1.195 (0.831-1.712)
0.41-0.50	1.713 (0.759-3.868)	1.659 (0.912-3.019)	2.086 (1.204-3.614)
>0.50	0.844 (0.445-1.601)	1.217 (0.705-2.100)	1.341 (0.907-1.981)
Adolescents			
0.31-0.40	0.824 (0.282-2.402)	1.451 (0.595-3.450)	1.582 (0.849-2.948)
0.41-0.50	0.604 (0.175-2.077)(	0.924 (0.400-2.132)	1.182 (0.601-2.322)
>0.50	0.852 (0.370-1.964)	4.822 (1.904-12.214)	3.059 (1.469-6.369)

^a^ Children are aged 6 to 11 years; adolescents, aged 12 to 15 years.

**Table 5.** Association Between Fluoride Exposure and Fluorosis Adjusting for Covariates

Fluoride exposure	Adjusted odds ratio (95% CI)^a^		
	2013-2014 Cycle	2015-2016 Cycle	2013-2016 Cycles
Fluoride supplement use			
No	1 [Reference]	1 [Reference]	1 [Reference]
Yes	0.561 (0.307-0.967)	0.741 (0.367-1.408)	0.727 (0.459-1.041)
Water fluoride level, mg/L			
≤0.30	1 [Reference]	1 [Reference]	1 [Reference]
0.31-0.50	1.626 (0.796-3.041)	1.105 0.377-3.469)	1.255 (0.594-2.692)
0.51-0.70	2.411 (0.856-6.587))	1.828 (0.735-4.909)	2.316 (1.260-4.390)
>0.70	2.333 (1.084-5.246)	2.378 (1.218-5.345)	2.790 (1.582-5.249)
Plasma fluoride level, μmol/L			
≤0.30	1 [Reference]	1 [Reference]	1 [Reference]
0.31-0.40	0.946 (0.506-1.981)	1.224 (0.848-1.833)	1.380 (0.995-2.029)
0.41-0.50	1.128 (0.521-2.860)	1.395 (0.785-2.633)	1.771 (1.032-3.116)
>0.50	0.856 (0.484-1.747)	1.568 (1.038-2.499)	1.659 (1.154-2.430)

^a^ Regression analyses were adjusted for age, sex, race and ethnicity, family educational level, ratio of family income to poverty, and 6-month period when surveyed.

## Discussion

In this cross-sectional study of a nationally representative population of US children and adolescents aged 6 to 15 years, we found that compared with the reference groups of 0.30 mg/L or less for fluoride water concentration and a plasma level of 0.30 μmol/L or less, higher levels of fluoride in plasma and water were independently associated with an increased risk of dental fluorosis. These findings are consistent with previous studies that found dental fluorosis might occur even with low levels of fluoride exposure from water.^18,19^ To reduce the effects of water fluoridation, the DHHS and policy makers may need to reconsider current recommendations for water fluoridation. In addition, it was not surprising that children who used fluoride supplements experienced lower water fluoride concentration exposures than those who did not take any fluoride supplements. It was reassuring that in this group, fluoride supplements were not associated with an increased risk of dental fluorosis, since these findings were not statistically significant. This finding may support the American Dental Association’s recommendation that children at high risk for cavities with low fluoride levels in their drinking water can safely benefit from fluoride supplements.^20^

Another key finding was that the overall prevalence of fluorosis for both the 2013-2014 cycle (87.3%) and 2015-2016 cycle (68.2%) was greater than the 23% prevalence reported in 2004 by the Centers for Disease Control and Prevention.^21^ While the prevalence may seem surprisingly high, it parallels an upward trend identified by Wiener et al,^22^ who reported an increase of 31.6% in fluorosis prevalence in adolescents aged 16 and 17 years between 2001 to 2002 and 2011 to 2012. Our results also align with those of Neurath et al,^23^ who found large increases in both the prevalence and severity of fluorosis over a 26-year period, peaking at a prevalence of 65% in 2011 to 2012. One reason for the increase in fluorosis prevalence may be the wider use of fluoride toothpaste and dental fluoride treatments. In contrast, 1 possible explanation for the decline in prevalence between the 2013-2014 and 2015-2016 cycles seen in this study may be the 2015 recommendation by the DHHS to lower water fluoride concentrations from 1.2 to 0.7 mg/L to minimize the risk of dental fluorosis.^12^ This policy change is also consistent with the lower plasma fluoride levels seen in the 2015-2016 group. However, the full effect of the 2015 recommendation may not be evident until later NHANES cycles since some 2015 enrollees may have been exposed to higher fluoride concentrations when their permanent teeth were forming. Additional studies examining whether this decline persists will be important for assessing the new recommendation’s impact on fluorosis.

The finding that well over half of the study group had some degree of fluorosis suggests that strategies to reduce the prevalence of dental fluorosis may be of value. However, when policies to reduce dental fluorosis are considered, the flip side is the potential loss of cavity protection. As policy makers weigh this balance, it should be noted that Do and Spencer^24^ did not find a negative association between mild dental fluorosis and the perception of dental appearance, self-rated oral health, or child or parent perceptions about their oral health. Similarly, another study^25^ reported no negative effects on oral health–related quality of life with mild fluorosis and even some suggestion of enhanced oral health–related quality of life with mild fluorosis.

### Strengths and Limitations

A strength of this study is its generalizability to the childhood population in the US. However, several limitations need to be considered. First, this study was cross-sectional rather than longitudinal, and while it demonstrates an association between fluoride exposure and fluorosis, this does not necessarily mean causation. Having a longitudinal study would allow for observation of the effect of fluoride over a longer period. Additionally, measuring fluoride levels in drinking water and plasma at a single time point might not accurately reflect exposure levels in the years when the permanent teeth of the participants were forming. The data for individuals who are ingesting tap water and were not reported may also contribute to the exposure levels. Receiving a fluoride supplement was a self-reported variable from the parents, and the use of questionnaires are subject to recall bias and misreporting. Furthermore, fluoride supplement use did not include information such as the length of use and the fluoride dose.

## Conclusions

In this cross-sectional study of 2995 participants using data obtained from the 2013-2014 and 2015-2016 NHANES cycles, exposure to higher concentrations of fluoride in water and having higher plasma fluoride levels were associated with a greater risk of dental fluorosis. In the 2013-2014 cycle, 87.3% of children exhibited some degree of dental fluorosis and 68.2% in the 2015-2016 cycle, a reduction that may be due to the 2015 DHHS recommendation to lower water fluoride concentrations. Further research is needed to assess the new fluoridation standard and to incorporate fluoride exposures from dietary fluoride supplements, topical fluoride application, fluoride toothpaste, fluoridated water, and natural products without fluoride to help policy makers balance caries prevention with dental fluorosis.

## Notes

Supplement 1. **eTable.** Association Between Fluoride Exposure and Fluorosis With Interaction Effect Adjusting for Covariates

Supplement 2.
Data Sharing Statement
