# Errors in Abstract, Results, Tables, and Supplement 1

**DOI:** 10.1001/jamanetworkopen.2024.11597

**Published:** 2024-04-10

The Original Investigation titled “A National Study Exploring the Association Between Fluoride Levels and Dental Fluorosis,”^1^ published June 23, 2023, contained erroneous 95% CIs in the Abstract, Results, tables, and Supplement 1 that resulted from inadvertent application of incorrect sample weight variables during the data analyses. Also, sample data were added to the weighted data given in Table 1 to align the report with different preferences for displaying descriptive statistics. The authors have explained what happened in a Comment.^2^ This article has been corrected.

## References

[1] Hung M, Hon ES, Mohajeri A, . A national study exploring the association between fluoride levels and dental fluorosis. JAMA Netw Open. 2023;6(6):e2318406. doi:10.1001/jamanetworkopen.2023.18406 37351888 PMC10290240

[2] Hung M. Response. Re: Potential statistical errors related to sampling design. *JAMA Network Open*. March 13, 2024. Accessed March 15, 2024. https://jamanetwork.com/journals/jamanetworkopen/fullarticle/2806509

