# Supplementary material for: A National Study Exploring the Association Between Fluoride Levels and Dental Fluorosis
Source: JAMA Netw Open. 2023 Jun 23;6(6):e2318406. doi: 10.1001/jamanetworkopen.2023.18406 (PMC10290240; doi:10.1001/jamanetworkopen.2023.18406)
Supplement: Supplement 1. — eTable. Association Between Fluoride Exposure and Fluorosis With Interaction Effect Adjusting for Covariates [file jamanetwopen-e2318406-s001.pdf]

Supplementary Online Content

Hung M, Hon ES, Mohajeri A, et al. A national study exploring the association between fluoride levels and dental fluorosis. *JAMA Netw Open*. 2023;6(6):e2318406. doi:10.1001/jamanetworkopen.2023.18406

eTable. Association Between Fluoride Exposure and Fluorosis With Interaction Effect Adjusting for Covariates

This supplementary material has been provided by the authors to give readers additional information about their work.

© 2023 Hung M et al. *JAMA Network Open*.

eTable. Association Between Fluoride Exposure and Fluorosis With Interaction Effect Adjusting for Covariates

Fluoride exposure	Fluorosis [a]		
	2013-2014	2015-2016	2013-2016
	AOR (95%CI)[b]	AOR (95%CI)[b]	AOR (95%CI)[b]
Fluoride supplement			
(Ref: No)			
Yes	0.639 (0.161-1.318)	0.762 (0.429-5.652)	0.901 (0.338-2.202)
Water fluoride level, mg/L			
(Ref: 0.00 - 0.30)			
0.31 - 0.50	1.336 (0.696-16.791)	1.067 (0.081-13.708)	1.169 (0.394-6.776)
0.51-0.70	2.680 (0.235-16.061)	1.551 (1.006-13.753)	2.139 (0.754-7.099)
> 0.70	2.513 (0.396-7.443)	2.058 (0.715-27.860)	2.541 0.806-7.587)
Plasma fluoride level, μmol/L			
(Ref: 0.00 - 0.30)			
0.31 - 0.40	0.789 (0.451-1.538)	1.172 (0.716-1.944)	1.181 (0.882-1.832)
0.41 - 0.5	0.811 (0.457-2.047)	1.190 (0.550-2.063)	1.319 (0.863-2.505)
> 0.50	0.545 (0.310-1.364)	1.241 (0.338-2.129)	1.141 (0.738-1.932)
Interaction effect			
Fluoride supplement* Water fluoride (0.31 - 0.50)	3.585 (0.216-5.938)	0.796 (0.441-11.487)	1.124 (0.352-3.133)
Fluoride supplement* Water fluoride (0.51 - 0.70)	0.618 (0.040-1.238)	2.016 (0.180-26.944)	1.005 (0.134-3.787)
Fluoride supplement* Water fluoride (> 0.70)	0.801 (0.108-8.318)	2.624 (0.242-3.089)	1.082 (0.253-3.223)

Note.
[a] Fluorosis: 0 = No fluorosis (DFI ≤ 0.5); 1 = Fluorosis (DFI ≥ 1).
[b] AOR = Adjusted Odds Ratio; CI = Confidence Interval; Regression analyses were adjusted for age, gender, race/ethnicity, family educational level, ratio of family income to poverty, and the time period when the survey was administered.

© 2023 Hung M et al. *JAMA Network Open*.
