# Supplementary material for: A National Study Exploring the Association Between Fluoride Levels and Dental Fluorosis
Source: JAMA Netw Open. 2023 Jun 23;6(6):e2318406. doi: 10.1001/jamanetworkopen.2023.18406 (PMC10290240; doi:10.1001/jamanetworkopen.2023.18406)
Supplement: Supplement 2. — Data Sharing Statement [file jamanetwopen-e2318406-s002.pdf]

Data Sharing Statement

Hung. A National Study Exploring the Association Between Fluoride Levels and Dental Fluorosis. *JAMA Netw Open*. Published June 23, 2023.
doi:10.1001/jamanetworkopen.2023.18406

Data
Data available: Yes
Data types: Deidentified participant data, Data (not involving human participants), Data dictionary
How to access data: https://wwwn.cdc.gov/nchs/nhanes/
When available: With publication

Supporting Documents
Document types: None

Additional Information
Who can access the data: Anyone requesting the data.
Types of analyses: For any purpose.
Mechanisms of data availability: With investigator support.
Any additional restrictions: None.
